# A Mini Zinc-Finger Protein (MIF) from *Gerbera hybrida* Activates the GASA Protein Family Gene, *GEG*, to Inhibit Ray Petal Elongation

**DOI:** 10.3389/fpls.2017.01649

**Published:** 2017-09-22

**Authors:** Meixiang Han, Xuefeng Jin, Wei Yao, Lingjie Kong, Gan Huang, Yujin Tao, Lingfei Li, Xiaojing Wang, Yaqin Wang

**Affiliations:** ^1^Guangdong Provincial Key Laboratory of Biotechnology for Plant Development, School of Life Sciences, South China Normal University Guangzhou, China; ^2^Key Laboratory of Southern Subtropical Plant Diversity, Fairy Lake Botanical Garden, Shenzhen and Chinese Academy of Sciences Shenzhen, China

**Keywords:** *Gerbera hybrida*, *GEG*, *GhMIF*, ray petal elongation, transcription factors

## Abstract

Petal appearance is an important horticultural trail that is generally used to evaluate the ornamental value of plants. However, knowledge of the molecular regulation of petal growth is mostly derived from analyses of *Arabidopsis thaliana*, and relatively little is known about this process in ornamental plants. Previously, *GEG* (*Gerbera hybrida* homolog of the gibberellin [GA]–stimulated transcript 1 [*GAST1*] from tomato), a gene from the GA stimulated Arabidopsis (GASA) family, was reported to be an inhibitor of ray petal growth in the ornamental species, *G. hybrida*. To explore the molecular regulatory mechanism of *GEG* in petal growth inhibition, a mini zinc-finger protein (MIF) was identified using yeast one-hybrid (Y1H) screen. The direct binding of *GhMIF* to the *GEG* promoter was verified by using an electrophoretic mobility shift assay and a dual-luciferase assay. A yeast two-hybrid (Y2H) revealed that GhMIF acts as a transcriptional activator. Transient transformation assay indicated that *GhMIF* is involved in inhibiting ray petal elongation by activating the expression of *GEG*. Spatiotemporal expression analyses and hormone treatment assay showed that the expression of *GhMIF* and *GEG* is coordinated during petal development. Taken together, these results suggest that *GhMIF* acts as a direct transcriptional activator of *GEG*, a gene from the GASA protein family to regulate the petal elongation.

## Introduction

Petals, as a component of the floral organs of angiosperms, play important roles in attracting pollinators, and in protecting stamens and pistils (Hermann and Kuhlemeier, 2011). Petal growth and final size are determined by coordinated cell division and expansion (Mizukami and Fischer, 2000; Szécsi et al., 2006), and in *Arabidopsis thaliana*, it has been shown that the early phases of petal growth depend on cell division, whereas later stages of flower opening are largely controlled by cell expansion (Varaud et al., 2011). These two stages are both regulated by environmental stimuli, hormonal signals and gene regulatory networks (GRNs) with the associated transcription factors (TFs) (Alvarez-Buylla et al., 2010; Ó’Maoiléidigh et al., 2014). In *A. thaliana, JAGGED, AINTEGUMENTA*, and *ARGOS*, which function as positive regulators, were all shown to affect petal growth by regulating cell division (Mizukami and Fischer, 2000; Hu et al., 2003; Dinneny et al., 2004). Other genes, such as *BIGBROTHER, KLUH* and *DA1* (DA means “large” in Chinese), were shown to affect final petal size by suppressing cell proliferation (Disch et al., 2006; Anastasiou et al., 2007; Li et al., 2008). *BIGPETALp (BPEp)*, a basic helix-loop helix (bHLH) TF, restricts cell expansion and petal size through interaction with the *AUXIN RESPONSE FACTOR8* (*ARF8*) (Szécsi et al., 2006; Varaud et al., 2011). Moreover, the expression of *BPEp* is also activated by jasmonate, providing another level of hormonal control on petal cell expansion (Brioudes et al., 2009). In horticultural plants, a CYCLOIDEA-like TCP domain TF, *GhCYC2*, is involved in the control of the identity and radial extent of flower types in *Gerbera hybrida* (Broholm et al., 2008). Subsequently more TCP transcription factors have been identified in *Senecio* (*RAY1,2,3*) and *sunflower* (*HaCYC2c*), which all play a specific role in the differentiation of ray and disk florets (Kim M. et al., 2008; Chapman et al., 2012; Garcês et al., 2016). *DgSZFP*, a C_2_H_2_-zinc finger TF, isolated from *chrysanthemum*, is reported to involve in floral organ development through increasing the width of petal tubes (Liu et al., 2014). While an ethylene-responsive NAC-domain transcription factor from rose (*Rosa* *hybrida*), *RhNAC100*, suppresses cell expansion during petal growth (Pei et al., 2013).

One of the large TF families in plants that are known to be involved in floral development, is the zinc finger domain containing protein (ZFPs) family (Cheuk and Houde, 2016). These proteins play important roles in many developmental processes, such as seed germination, floral organ identity specification, plant size and flowering, cell elongation, secondary cell wall formation and anther development (Li et al., 2001; Kim D.H. et al., 2008; Kim et al., 2014; Chai et al., 2015; Lin et al., 2015). In addition to the ZFPs, a novel putative zinc-finger protein was recently defined (Baek et al., 2015). Due to their small size (approximately 100 amino acid residues), these proteins have been named MINI ZINC FINGER (MIF) proteins (Sicard et al., 2008). In *A. thaliana*, three MIF proteins, AtMIF1, AtMIF2, and AtMIF3, have been identified. Constitutive expression of *AtMIF1* inhibits the development of floral organs, reducing the size of petals, sepals, and stamens through suppression of cell elongation and expansion (Hu and Ma, 2006). The expression pattern of *AtMIF3* is similar to that of *AtMIF1*, and overexpression of *AtMIF3* was shown to disrupt determinate leaf growth by inducing ectopic shoot meristems on leaf margins (Hu et al., 2011). The regulatory role of *AtMIF2* has not yet been determined. In tomato (*Solanum lycopersicum*), *INHIBITOR OF MERISTEM ACTIVITY* (*IMA*) was identified as a mini zinc-finger gene with high similarity to *AtMIF2*, and shown to act as a regulator of meristem activity during flower and ovule development (Sicard et al., 2008).

Another protein family that is involved in floral development is the GASA [gibberellin (GA) stimulated Arabidopsis] family, members of which contain a conserved C-terminal region of approximately 60 amino acids, with 12 Cys residues at conserved sites and a putative N-terminal signal peptide for targeting to the secretory pathway (Roxrud et al., 2007). They have been shown to be involved in petal elongation, regulation of plant growth and fruit size, shoot elongation and flower transition, flowering and stem growth, and modulation of the brassinosteroid and gibberellin signaling pathways (Roxrud et al., 2007). *GAST1* (the GA-stimulated transcript 1) was first identified as a GA-stimulated gene in tomato (Shi et al., 1992), and subsequently numerous *GAST1* homologs are also found in various plant species (Taylor and Scheuring, 1994; Kotilainen et al., 1999; Segura et al., 1999; Bennissan et al., 2004; Fuente and Valpuesta, 2006; Furukawa et al., 2006).

*Gerbera hybrida*, a member of the Asteraceae family, has a unique inflorescence structure with three special types of florets including the outermost ray florets, the middle trans florets and the inner disk florets (Laitinen et al., 2005), and the size and shape of the petal is genetically determined (Laitinen et al., 2007). Petals of outer ray flowers are long and bilaterally symmetrical, whereas those of disk flowers are short and radially symmetrical in the centermost of the capitulum (Laitinen et al., 2005). These characteristics make it an interesting model system to study petal growth and development. Two *GASA* genes from *G. hybrida* that play roles in petal development have been identified: *PRGL* (Proline-rich and GASA-like), has a higher expression level during early development in ray petals (Peng et al., 2008, 2010), while *GEG* (Gerbera homolog of *GAST1* gene), was shown to inhibit the growth of ray floret corolla by reducing cell length during the later development stages (Kotilainen et al., 1999). Although a number of candidate genes with putative regulatory roles have been found to be involved in petal organogenesis in *G. hybrida* (Laitinen et al., 2007), the molecular mechanism by which *GEG* inhibits petal elongation remains unknown.

Here, a MIF from *G. hybrida*, GhMIF, was identified through a yeast one-hybrid (Y1H) screening system, as a protein interacting with the core region of the *GEG* promoter. A range of molecular and genetic technologies were then used to examine its regulatory role and association with *GEG* to inhibit ray petal elongation. Moreover, expression analysis showed that the expression of *GhMIF* and *GEG* is coordinated during petal development. Therefore, our work presented a new picture in which the *GhMIF*/*GEG* module plays an important role in petal growth of *G. hybrida*.

## Materials and Methods

### Plant Materials and Growth Conditions

*Gerbera hybrida “*Shenzhen No. 5” seedlings were grown under standard greenhouse conditions at 26/18°C (day/night temperature) and a relative humidity of 65–80%. Ray petals at different developmental stages (Meng and Wang, 2004), and other tissues and organs were sampled for the following experiments.

*Arabidopsis thaliana* seeds (Col-0) were surface sterilized, plated on Murashige and Skoog medium (MS) (Sigma–Aldrich), and imbibed in darkness at 4°C for 3 days for vernalization. The plates were transferred to a growth room (21–22°C, 60% relative humidity) under long-day conditions (16 h light/8 h dark) for 7 days, then seedlings were transplanted into pots containing peat:vermiculite (3:1) and grown under the same conditions.

### Cloning and Bioinformatic Analysis of the *GEG* Promoter

The promoter sequence of *GEG* was obtained by high-efficiency thermal asymmetric interlaced PCR (Hi-TAIL PCR) as previously described (Liu and Chen, 2007). The promoter sequence was analyzed using PLACE^1^ and PlantCARE^2^. The primers used for this experiment are listed in Supplementary Table S1.

### Protoplast Transformation and Dual-Luciferase Reporter Assay

Protoplasts were isolated from the rosette leaves of 4-week-old Col-0 as previously (Sun et al., 2013). The dual-luciferase assay was performed according to (Hellens et al., 2005). To characterize the core *GEG* promoter sequences, four fragments of the *GEG* promoter, named P1365, P880, P580, P260 (with the numbers representing the base pairs to the start of each fragment upstream from the ATG translational start site) were fused into pGREEN0800-LUC to generate reporter vectors. The modified pBluescript vector (pBS) (Paul et al., 2016) was used as an effector. For the interaction study of *GhMIF* and the *GEG* promoter, the pGREEN0800-LUC plasmid incorporated with *pGEG_320_* (-580∼-261) was used as the reporter, and the *pBS-GhMIF* was used as an effector. Meanwhile, the pGREEN0800-LUC plasmid incorporated with *pGEG_170_* (-430∼-261) was used as the unspecific binding control. The effectors were co-introduced with the reporters into the protoplasts as previously described (Yoo et al., 2007) and the transformed protoplasts were incubated at room temperature for 20–22 h. The dual-luciferase assay was performed as described by the manufacturer (Dual-Luciferase^®^ Reporter Assay, Promega, United States), and the Firefly and Renilla luciferase activities were detected using an Enspire multi-mode microplate reader (PerkinElmer Inc., United States). Three biological replicates were performed for all experiments.

### cDNA Library Construction and Yeast One-Hybrid (Y1H) Screen

For the preparation of cDNA library of *G. hybrida*, total RNA was extracted from ray petals at stage 1 to 6 (S1–S6) (Meng and Wang, 2004) with a RNA extraction kit (Waryong, Beijing) according to the manufacturer’s instructions. A cDNA library of a storage capacity of 1.05 × 10^7^ CFU was established after a series of steps: mRNA purification, cDNA synthesis, 5′ adaptor ligation, homogenization treatment, fusion with AD vector and transformation to *Escherichia coli*. To obtain the interaction proteins with *pGEG_320_*, the cDNA library was introduced into bait strains carrying the pGEG_320_-AbAi vectors. Screening and identification of interaction colonies were carried out as described in the Matchmaker Gold Yeast One-Hybrid Library Screening System kit (Clontech, United States). GhMIF, a potential interaction protein, was selected for further analysis.

To confirm the interaction between the *pGEG_320_* and GhMIF, the *pGEG_320_* region (from -580 to -261 bp upstream of the initial codon ATG) and *pGEG_170_* (-430∼-261) were inserted into the pAbAi vector, which was linearized by BstBI digestion and transformed into a Y1HGold strain to generate the Y1H bait strain and the *GhMIF* ORF (309 bp) was inserted into the pGADT7 vector as the prey plasmid. Y1HGold transformed with pGEG_320_-AbAi was grown in SD-Ura medium to screen for successful transformation, then the pGADT7 prey vector harboring GhMIF was integrated into Y1HGold [pBait-AbAi] yeast strains, and the yeast cells were grown on SD-Leu medium with 200 ng/mL AbA (Aureobasidin A, Clontech, United States) to test the interaction. The plates were cultured at 30°C for 3–5 days. Primers used for the Y1H assay are listed in Supplementary Table S1.

### Yeast Two-Hybrid (Y2H) Assay

A Y2H assay was performed using a Clontech (Matchmaker^®^ Gold Yeast Two-Hybrid System, Cat. No. 630489) kit with the AH109 yeast strain. The *GhMIF* ORF was sub-cloned into the *Eco*RI/*Bam*HI sites of the pGBKT7 vector (Gal4 DNA binding domain, Clontech) as the bait. The bait construct and empty pGADT7 (Gal4 activation domain, Clontech) were co-transformed into AH109 as previously described (Gietz and Schiestl, 2007). Transformed monoclonal yeast cells were identified on SD-Trp/-Leu medium and transferred to SD-Leu/-Trp/-His medium containing 20 mg/ml 5-Bromo-4-chloro-3-indolyl-D-galactopyranoside (X-Gal, Clontech) to test the transcription activation activity and images were acquired after incubation at 30°C for 3 days. Primers used for the Y2H assay are listed in Supplementary Table S1.

### Electrophoretic Mobility Shift Assay (EMSA)

Recombinant pET28a-SUMO-GhMIF proteins were expressed in *E. coli* BL21 cells induced by the addition of 0.5 mM isopropyl-β-D-thiogalactopyranoside (IPTG) overnight at 22°C, and purified on Ni-NTA columns (Qiagen, Germany). The core region of the *GEG* promoter *pGEG_320_* (-580∼-261) was used as a 5′ end biotin labeled probe using T4 polynucleotide kinase and the same fragment, but unlabeled, was used as a competitor. Meanwhile, *pGEG_150_* (-580∼-431) and *pGEG_170_* (-430∼-261) from the truncation of *pGEG_320_* (-580∼-261) were used as 5′ end biotin labeled probes for specific interaction verification. An EMSA analysis was conducted using the LightShift Chemiluminescent EMSA kit (Thermo Scientific, United States). After incubation at room temperature for 20 min, the reaction mixture containing 1 μg of purified fusion protein and 50 nmol/mL biotin-labeled probe for the binding reaction were electrophoresed on a 6% polyacrylamide mini-gel, and then transferred onto a positively charged nylon membrane (GE Healthcare, United States) and the transferred DNA cross-linked to the membrane with an ultraviolet lamp. The primers used for EMSA are listed in Supplementary Table S1.

### Subcellular Localization Assay

*GhMIF* was amplified and cloned into the modified vector pBluescript II SK carrying a yellow fluorescence protein (YFP) to generate the *YFP-GhMIF* construct. The construct was then transformed into *A. thaliana* protoplasts (generated as above) and the fluorescence was detected with a laser confocal scanning microscope (LSM710, CarlZeiss, Germany) approximately 12 h after transformation. The empty YFP vector was used as a control. Primers used for the experiment are listed in Supplementary Table S1.

### Transient Transformation of Ray Petals

A transient petal transformation assay was performed as previously described (Pei et al., 2013). The *GhMIF* ORF (309 bp) was used to generate an overexpression vector (*pCANG-GhMIF*) which is derived by the 35S promoter and a virus-induced gene silencing vector (*pTRV2-GhMIF*). *pTRV2-GhMIF, pTRV1* and *pCANG-GhMIF* were separately transformed into *Agrobacterium tumefaciens* strain C58C1. *A. tumefaciens* was grown in Luria-Bertani broth (LB) containing 75 μg mL^-1^ kanamycin and 60 μg mL^-1^ rifampicin at 28°C with shaking at 200 rpm overnight. The cultures were then diluted 1:50 (v/v) in fresh LB containing 10 mM of 2-morpholinoethanesulfonic acid (MES), 20 mM acetosyringone, 75 μg mL^-1^ kanamycin and 60 μg mL^-1^ rifampicin and grown overnight (12–14 h). The cultures were centrifuged at 1,000 *g* for 10 min and resuspended in an infiltration buffer (10 mM of MES, 10 mM of MgCl_2_, 20 mM acetosyringone, pH = 5.6) to an OD_600_ of ∼1.2. *A. tumefaciens* cultures containing *pTRV2-GhMIF* and *pTRV1* at a ratio of 1:1 (v/v), and a mixture containing pTRV2/pTRV1 as a negative control, were stored at 28°C for 4 h in the darkness prior to infiltration.

Detached ray petals at stage 3 in length of 1.5–2.0 cm (Meng and Wang, 2004) were submerged into infiltration solution with a vacuum of -0.09 MPa for 5 min, then the vacuum was slowly released within 2 min to ensure *A. tumefaciens* entering ray petals. After infiltration, ray petals were rinsed with sterile distilled water (ddH_2_O) to remove the remnants and then placed in the glass dish with filter papers. After incubation at 8°C for 3 days, the transformed petals were transferred to a growth chamber at 23–25°C for 8 days with 50–60% humidity under long-day conditions (16 h light/8 h dark) and sprayed 1 mL ddH_2_O once a day. For each treatment, at least 90 petals with three technical replicates were infiltrated and three biological replicates were performed for the experiment. The sequences of the primers used are listed in Supplementary Table S1.

### Measurement of Ray Petal Length and Cell Size

Ray petal and cell lengths were measured as previously described (Li et al., 2015). After transient transformation, petals from different treatments (mock, *35S::GhMIF*, and *pTRV2-GhMIF/pTRV1*) were cultivated in normal growth conditions for 8 days. And photographs were taken each day with a digital camera (Nikon, D7200, Japan). A total of 90 petals for each treatment with three technical replicates were analyzed using ImageJ software^3^ (NIH, Bethesda, MD, United States) and data were used to evaluate the elongation rate of the basal and whole petals. The elongation rate was calculated according to the following equation: Elongation rate = (L*t*–L*i*)/L*i* × 100%, where L*t* is the petal length at the 8th day after normal cultivation while L*i* is the initial petal length at the 1st day after transferred to the normal growth conditions. For measurements of epidermal cell length and number, the basal regions of 10 petals from each treatment were stained with propidium iodide (0.1 mg mL^-1^) for 5 min at room temperature, then immediately washed with deionized water. Images of the epidermal cells were taken using a laser confocal scanning microscope (LSM710, CarlZeiss, Germany). To determine the average cell length, 20 epidermal cells from each photograph were measured using ImageJ. To determine the mean cell number, all epidermal cells in the visual field were counted. The average cell number was used to represent the total number of epidermal cells in each petal.

### Quantitative RT-PCR (qRT-PCR)

The tissue samples of *G. hybrida* were lyophilized and stored at -80°C until use. Total RNA was extracted with a RNA extraction kit (Waryong, Beijing) according to the manufacturer’s instructions. Extracted RNA was treated with DNase to remove contaminating DNA and the gene specific primers were designed by the Primer Premier 5. The first-strand cDNA was synthesized from the 0.5 μg of total RNA using the 5 × RT Master Mix (Toyobo, Japan). The synthesized cDNA (10 μl) was diluted into 50 μl with ddH_2_O and then used as template for qRT-PCR with the 2 × Realstar Green Fast Mixture (GeneStar, Beijing). Each PCR reaction (20 μl) contained 0.4 μl cDNA template, 10 μl 2 × RealStar Green Mixture, 0.4 μl (0.2 μM) gene-specific primers and 8.8 μl ddH_2_O. qRT-PCR was performed on a Bio-Rad CFX96^TM^ qRT-PCR system using the following procedure: 95°C for 60 s, followed by 40 cycles of 95°C for 15 s, 60°C for 15 s and 72°C for 45 s. Melt curve analysis was performed on the end of PCR using the following procedure: 65°C for 5 s, then to 95°C with the increment of 0.5°C/s, to determine the specificity of reactions. The PCR amplification efficiency of *ACTIN, GhMIF* and *GEG* is 93.5, 94.8, and 98.2%, respectively. The relative expression levels were calculated using the 2^-ΔΔC_T_^ method (Livak and Schmittgen, 2001). All reactions were performed with three technical and three biological replicates. The data were normalized to the *ACTIN* (AJ763915) gene as previously described (Kuang et al., 2013). The primers used are shown in Supplementary Table S1.

### Hormone Treatments of Ray Petals

Ray petals detached from inflorescences at stage 5 were placed on two layers of Whatman filter papers that were immersed in GA_3_ (10 μM), ABA (50 μM), GA biosynthesis inhibitor paclobutrazol (PAC) (10 μM) and ABA biosynthesis inhibitor fluridone (FLU) (0.1 μM), respectively, for 0, 4, 8, 12, 16, and 24 h at 23°C with 50–60% humidity under the long-day condition (16 h light/8 h dark). Petals treated with 0.1% ethanol in deionized water were used as a control. Approximately 0.1 g petal tissue from the different treatments for each time point were sampled in parallel then frozen in liquid nitrogen prior to processing for qRT-PCR analysis. Hormones and inhibitors were acquired from the Sigma–Aldrich Chemical Co. (Shanghai, China). The experiment was performed with three biological replicates.

### Statistical Analysis

One-Way ANOVA was used to analyze the data from the three biological replicates with SPSS 13.0 software (SPSS Inc., Chicago, IL, United States). Tukey’s honestly significant difference (HSD) test was applied to evaluate the statistical significance (^∗^*p* < 0.05, ^∗∗^*p* < 0.01).

## Results

### Cloning and Activity Analysis of *GEG* Promoter

*GEG* (AJ005206) from *G. hybrida* has been identified as a suppressor of ray petal growth in the later developmental stages (Kotilainen et al., 1999). However, the underlying molecular regulatory mechanism is not clear. Here, we first cloned 1,365 bp of the *GEG* promoter region by Hi-TAIL PCR (Supplementary Figure S1A), and showed by histochemical staining of *P1365::GUS* transgenic Arabidopsis seedlings that *GEG* expression was induced by GA (Supplementary Figure S1B) as previously reported (Kotilainen et al., 1999).

**FIGURE 1:**
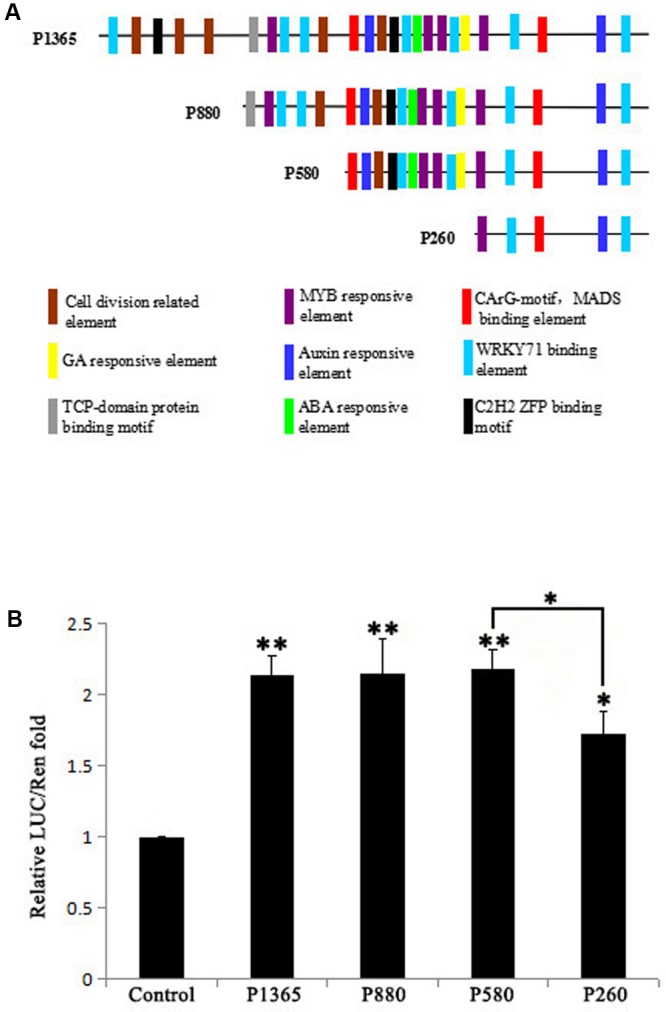
Bioinformatic prediction of transcription factor binding sites (TBS) in the *GEG* promoter and experimental verification of the core region that affects expression. **(A)** A series of 5′-truncated *GEG* promoter fragments (P1365, P880, P580, and P260) and the distribution of predicted TBS. The length of the fragments is indicated on the left side of each fragment. **(B)** Relative reporter activity of each promoter fragment in a dual-luciferase assay. Values are the means ± SD from three biological replicates. The LUC/REN fold value of the control was set to 1.0. The significant difference was analyzed by Tukey’s HSD: ^∗^*p* < 0.05, ^∗∗^*p* < 0.01.

*GEG* promoter was analyzed using PLACE^4^ and PlantCARE^5^ and the result showed that there distribute hormones (GA, ABA, and Auxin) responsive elements, binding elements for transcription factors (MYB, MADS, WRKY71, C_2_H_2_ ZFP, and TCP-domain protein) and cell division related elements in the 1,365 bp promoter (**Figure 1A**). To identify the core region in the 1,365 bp promoter that influence the expression level of *GEG*, four 5′ truncated fragments (P1365, P880, P580, and P260) of the *GEG* promoter (**Figure 1A**) were used to generate reporter constructs for a dual-luciferase assay in *A. thaliana* Col-0 protoplasts. The result showed that P1365, P880, and P580 all exhibited similar LUC/REN ratios (2.14- to 2.19-fold), which were significantly different from the control. However, P260 showed much lower reporter activity than P580 although it was higher than the control (1.73-fold) (**Figure 1B**). These data indicated that the region from -580 to -261 bp (*pGEG_320_*) in the *GEG* promoter is of importance for *GEG* expression, which was also supported by the results from the transient expression assay in rice callus (Supplementary Materials and Methods and Figure S1C).

### GhMIF Acts as a Transcription Activator of *GEG* by Direct Binding to Its Promoter

**FIGURE 2:**
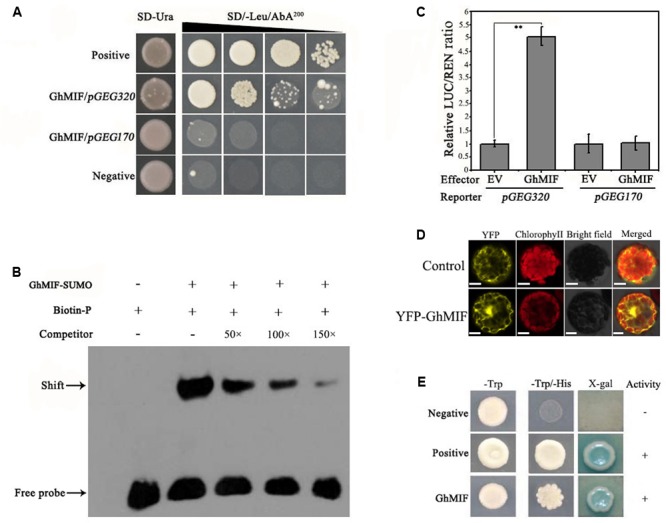
GhMIF acts as a direct activator of GEG transcription. **(A)** Yeast one-hybrid analysis showing the interaction between GhMIF and the GEG promoter. pGADT7-Rec-p53/p53-AbAi and pGADT7/pGEG_320_ were used as positive and negative controls, respectively. GhMIF/pGEG_170_ was used as the unspecific binding control. The black triangle indicates a gradient of bacterium concentration: 10^0^, 10^-1^, 10^-2^, and 10^-3^ from the left. **(B)** Electrophoretic mobility shift assay (EMSA) analysis showing the binding of GhMIF to the GEG promoter in vitro. The black arrow indicates the binding of GhMIF to the biotin-labeled GEG promoter. The + and – represent the presence and absence of corresponding components, respectively. **(C)** Dual-luciferase (LUC) assay indicating the interaction between GhMIF and the GEG promoter in vivo. As the unspecific binding control, the GhMIF/pGEG_170_ exhibited the similar LUC/REN ratio with the control (EV/pGEG_170_). The relative LUC activity of the control was set to 1.0. Values are the means ± SD from three biological replicates. ^∗∗^Indicates the significant difference (*p* < 0.01) by Tukey’s HSD test. **(D)** Subcellular localization of GhMIF in *Arabidopsis thaliana* protoplasts. Scale bar = 10 μm. **(E)** Transcriptional activation of GhMIF in yeast cells. *GhMIF* was fused to the yeast GAL4 DNA-binding domain, and its ability to activate the His or LacZ reporter gene was evaluated in yeast. The vectors pGBKT7/pGADT7 and pGBKT7-53/ pGADT7-Rec T were transformed into yeast cells and used as negative and positive controls, respectively.

To identify TFs that interact with the *GEG* promoter, a Y1H screen was performed (Supplementary Figures S2A,B). Using the core *GEG* promoter region *pGEG_320_* (-580 to -261 bp) as a bait, an interacting mini zinc-finger protein (MF370885, GhMIF) was identified, which shares 69% sequence identity with the AtMIF2 protein from *A. thaliana* (Supplementary Table S2). To confirm the interaction between GhMIF and the bait, a Y1H assay was performed, which further indicated that GhMIF could induce the expression of the reporter gene driven by the *GEG* promoter (**Figure 2A**), and suggested that GhMIF specifically binds to the *GEG* promoter.

We then performed an EMSA analysis to validate the physical interaction between GhMIF and the *GEG* promoter *in vitro*. The core *GEG* promoter region *pGEG_320_* (-580 to -261 bp) was used as a biotin-labeled probe, and the same oligonucleotide, but unlabeled, was used as a competitor. The competition assay was carried out by adding excess amounts of the unlabeled probe. As shown in **Figure 2B**, the GhMIF protein bound to the DNA probes. Furthermore, increasing the concentration of unlabeled probe in the binding reactions resulted in weaker bands, suggesting that the GhMIF protein directly bound to the core region of the *GEG* promoter *in vitro*. The truncation assay further found that GhMIF protein binds to the *pGEG_150_* region that contains the C_2_H_2_ ZFP binding motif (TACAAT), not to the *pGEG_170_* (Supplementary Figure S3), which suggested that GhMIF has a specific binding to *GEG* promoter.

We also performed a dual-luciferase assay with *A. thaliana* Col-0 leaf protoplasts. The recombinant *pGREEN0800-GEG_320_* and *pGREEN0800-GEG_170_* as reporters, respectively, and the fused protein with GhMIF as an effector, were co-introduced into the protoplasts. Co-transformation of *pGEG_320_* and the GhMIF protein resulted in a higher LUC/REN ratio compared with the control, but the LUC/REN ratio for *pGEG_170_* and GhMIF protein was similar to the control (**Figure 2C**). The result indicated that GhMIF specifically binds to the *GEG* promoter *in vivo* and can activate the expression of the reporter gene. Meanwhile, we performed a subcellular localization analysis of GhMIF and a transactivation assay using a Y2H assay. The results showed that the YFP-GhMIF fusion protein accumulated in both the nucleus and cytoplasm (**Figure 2D**) and GhMIF acts as a transcriptional activator by the detection of β-glucosidase activity (**Figure 2E**). Taken together, these data indicated that GhMIF acts as a transcriptional activator of *GEG*.

### GhMIF Inhibits Ray Petal Elongation by Activating *GEG* Expression

**FIGURE 3:**
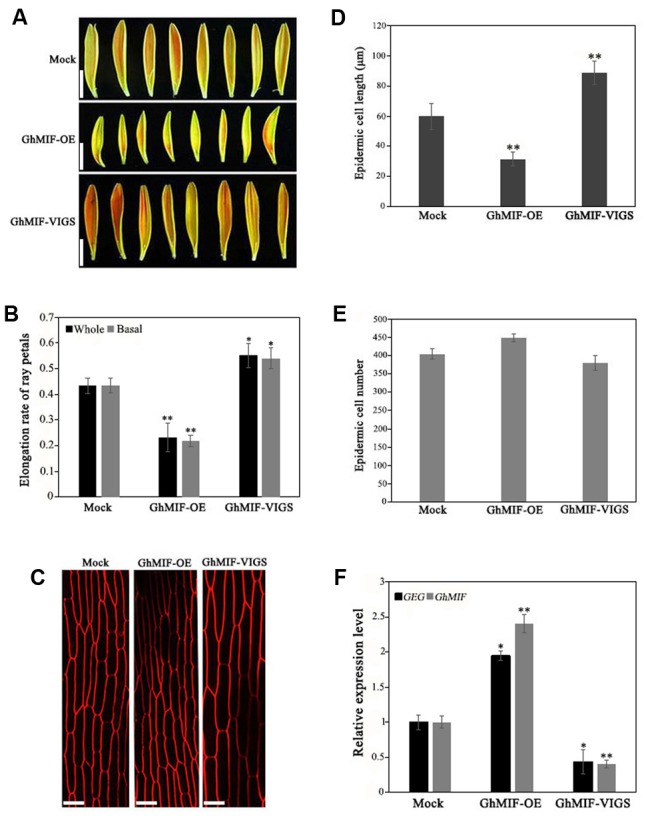
Overexpression and silencing of *GhMIF* in *Gerbera hybrida* petals. **(A)** Ray petal phenotypes and determination of **(B)** the whole and basal ray petal elongation rate. **(C)** Epidermal cell images of mock controls, *GhMIF-OE* and *GhMIF-VIGS* lines. **(D)** Cell length and **(E)** cell number of mock controls, *GhMIF-OE* and *GhMIF-VIGS* lines. **(F)** The expression level of *GhMIF* and *GEG* in mock control, *GhMIF-OE* and *GhMIF-VIGS* petals. The experimental petals were collected after 8 days cultivation in growth conditions. All values indicate means ± SD from at least three biological replicates. Tukey’s HSD: ^∗^*p* < 0.05, ^∗∗^*p* < 0.01. Scale bars represents 1 cm **(A)** or 20 μm **(C)**.

To characterize the function of *GhMIF* in petal growth, transient transformation assays of ray petals were performed. After transformation and incubation at 8°C for 3 days, ray petals were transferred to a growth chamber at 23–25°C for 8 days. From days 1 to 8, the elongation rate in petals over-expressing *GhMIF* (*35S::GhMIF*) was lower than in control petals, but was higher in petals in which *GhMIF* expression was suppressed using virus induced gene silencing (VIGS) (*pTRV2-GhMIF/pTRV1*). For the whole petals (including top, middle, and basal regions), the elongation rates of ray petals were 0.23 ± 0.06 in *35S::GhMIF* and 0.55 ± 0.05 in *pTRV2-GhMIF/pTRV1*, which corresponds to a decrease of 47% and an increase of 27%, respectively, compared with the elongation rate of 0.43 ± 0.03 in the mock control (**Figures 3A,B**). Because the basal region of ray petals was the main zone of elongation during treatment with GA3 (Li et al., 2015), we then focused on this area. As shown in **Figure 3B**, the elongation rates of basal petals were 0.22 ± 0.02 in *35S::GhMIF* and 0.54 ± 0.04 in *pTRV2-GhMIF/pTRV1*, with a decrease of 50% and an increase of 24%, respectively, compared with that of the mock control.

To determine whether the change in petal length was caused by cell elongation or cell division, we measured the number and size of the basal cells in ray petals after cultivation for 8 days. As shown in **Figures 3C,D**, the average length of the epidermal cells was 32 μm in *35S::GhMIF* petals compared with 60 μm in the mock controls. In contrast, the average length of the epidermal cells from *pTRV2-GhMIF/pTRV1* lines was approximately 89 μm. No significant differences in cell numbers were observed in the transiently transformed petals (**Figure 3E**). The data suggested that *GhMIF* inhibits petal elongation primarily by affecting cell elongation, rather than cell division.

Quantitative RT-PCR assays were also performed to detect the expression level of *GhMIF* and *GEG* in the transient transformation lines (Supplementary Figure S4). The result showed that the expression level of *GhMIF* was significantly upregulated (∼2.40-fold) in the overexpression lines and downregulated (∼0.40-fold) in the silenced lines compared with the mock control (**Figure 3F**), which indicated that the *GhMIF* expression level was inversely proportional to the petal elongation rate and cell length (**Figures 3B–D**). Besides, the *GEG* expression was significantly upregulated (∼1.95-fold) in petals of the *GhMIF* overexpression lines, but decreased (∼0.43-fold) in silenced lines, compared with the mock control (**Figure 3F**). Taken together, these results showed that *GhMIF* inhibits petal growth by activating the expression of *GEG*.

### GhMIF Is Involved in Petal Growth via a Coordinated Expression with *GEG*

**FIGURE 4:**
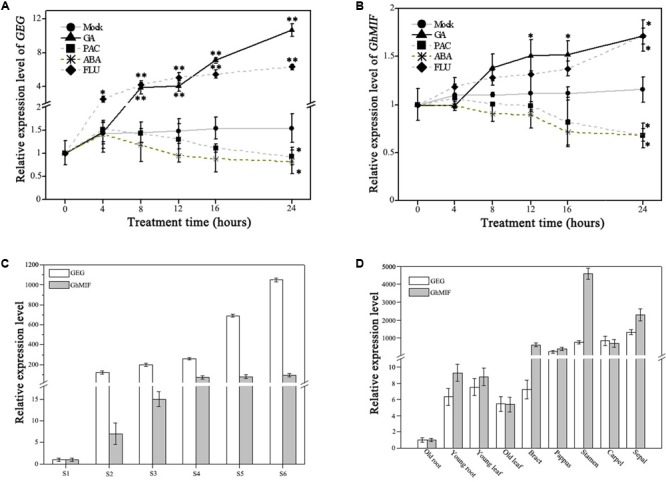
Expression analysis of GEG and GhMIF by qRT-PCR. Detection of the expression level of **(A)** GEG and **(B)** GhMIF in S5 ray petals under GA (gibberellic acid, 10 μM), ABA (abscisic acid, 50 μM), PAC (paclobutrazol, 10 μM), FLU (fluridone, 0.1 μM) and mock treatments at different time points (0, 4, 8, 12, 16, and 24 h). **(C)** Relative expression level of GEG and GhMIF during petal developmental phases (S1–S6, “S” represents “stage”) and **(D)** in different tissues and floral organs of the ray petals. GhACTIN (AJ763915) was used as an internal control. The experiment was repeated three times, the lowest expression in any tissue was set to 1.0, and the expression value indicates mean ± SD. The significant differences were evaluated by Tukey’ HSD test: ^∗^*p* < 0.05, ^∗∗^*p* < 0.01.

Previously GA was reported to activate the expression of *GEG* in ray petals and regulate petal growth antagonistically with ABA in *G. hybrida* (Kotilainen et al., 1999; Li et al., 2015). We evaluated the expression levels of *GhMIF* and *GEG* following treatments with GA, ABA and their respective biosynthetic inhibitors, PAC and FLU, using qRT-PCR. The expression levels of *GEG* and *GhMIF* increased after GA_3_ treatment and decreased in response to PAC treatment over the course of times. While ABA treatment attenuated and FLU treatment enhanced the expression of *GEG* and *GhMIF* (**Figures 4A,B**).

As shown in **Figure 4A**, the expression of *GEG* was upregulated significantly after 8 h and 4 h treatment with 10 μM GA_3_ and 0.1 μM FLU, respectively, compared with the mock control. Although the expression trend of *GhMIF* in response to GA_3_ and FLU was similar to that of *GEG, GhMIF* increased to a lesser extend (**Figure 4B**). In contrast, the expression of *GEG* was slightly suppressed from 0 to 16 h treated with 10 μM PAC and 50 μM ABA, which was in accordance with that of *GhMIF*. These results suggested that GA, ABA and their biosynthetic inhibitors affected the expression of *GEG* and *GhMIF* in a similar manner, but to a different extent.

Meanwhile, the expression level of *GEG* and *GhMIF* was investigated in the petal developmental stages (S1–S6) and different floral organs and tissues. The spatial–temporal expression assay showed that the expression level of *GEG* increased in parallel with the expression of *GhMIF* from S1–S6 (**Figure 4C**), and these two genes were expressed at higher levels in floral organs than in other tissues (**Figure 4D**). Taken together, these data are consistent with the expression of *GEG* and *GhMIF* being coordinated during petal development, which is also supported by the fact that the expression level of *GEG* was up-regulated and suppressed significantly in *GhMIF* over-expression and silenced lines, respectively (**Figure 3F**).

## Discussion

### The Mini Zinc-Finger Protein GhMIF Inhibits Ray Petal Elongation in *G. hybrida*

Petal growth is a complex physiological process that is regulated by complicated GRNs. Previous studies have suggested that zinc finger proteins are important components of GRNs. For example, in *A. thaliana*, a C_2_H_2_-zinc finger transcription factor, *RABBIT EARS* (*RBE*), is required for petal development of Arabidopsis, and its action results in the transition from cell division to post-mitotic cell expansion through repression of the *CIN-TCP* genes (*TCP5, TCP13, TCP17*) during early petal development (Huang and Irish, 2015). Meanwhile, in the Asteraceae plants, the *CYC* genes encoding TCP transcription factors have a conserved function in control of the identity of flower type, probably through cell division, cell elongation or organ fused events (Broholm et al., 2008; Kim M. et al., 2008). In addition, in *Chrysanthemum morifolium* ‘jinba,’ transcriptome and hormone analyses on petals revealed that some zinc finger proteins exhibit obvious up-regulation; a result that is congruent with them influencing petal growth (Wang et al., 2017). Mini zinc-finger proteins (MIF), a subgroup of the ZFP family, have been identified from a few species, namely Arabidopsis and tomato so far, and some of them were found to be involved in the development of floral organs and leaves, consistent with the functions of ZFPs family (Cheuk and Houde, 2016). In respect to the function of MIFs in petal growth, only AtMIF1 has been reported to inhibit the development of floral organs. However, the target genes of MIF or other MIF proteins still need to be explored (Hu and Ma, 2006).

In this study, for the first time we present the experimental evidences that GhMIF, a member of mini zinc-finger protein family, inhibit petal elongation by activating *GEG* in *G. hybrida*. This is supported by the following results: (1) For the whole petal, the elongation rate was decreased by 47% in *GhMIF* overexpression lines and increased by 27% in *GhMIF* silenced lines, compared with the mock control (**Figure 3B**), indicating that GhMIF is a negative regulator of petal elongation. Furthermore, our work also revealed that the basal region is the main elongation part of ray petals (**Figure 3B**), in agreement with previous studies (Li et al., 2015; Wang et al., 2017). (2) It was verified that GhMIF bound directly to the core region of the *GEG* promoter (**Figures 2A,B**). Moreover, the expression of *GEG* was significantly upregulated in petals of the *GhMIF* overexpression lines and decreased in those of *GhMIF* silenced lines, compared with the mock control (**Figure 3F**), which suggested that GhMIF could activate the expression of *GEG* consistent with the result in **Figure 2C**.

It’s also well known that petal growth is a complex process that integrates the cell division and cell expansion. And previous studies have revealed that petal size mainly depend upon cell expansion at the later stage of flower opening in *G. hybrida* (Meng and Wang, 2004; Zhang et al., 2012). In our work, GhMIF was confirmed to limit the cell expansion thereby affecting the petal size (**Figure 3C**), in which only the cell length is significantly changed, but the cell number remains unchanged compared with the mock control (**Figures 3D,E**) as previously described (Laitinen et al., 2007). Taken together, GhMIF plays important roles in the growth of ray petals by affecting cell elongation. Although some transcription factors with putative specificity to individual flower types have been identified by a 9k gerbera cDNA microarray, it is found that in disk flowers there only existed several basic TFs related to chromatin assembly, regulation of transcription initiation and mRNA processing (Laitinen et al., 2006). So, whether GhMIF is involved in the growth of disk florets remains to be investigated through various experimental methods, such as the RNA-blot and *in situ* hybridizations.

### GhMIF Is Involved in Petal Growth by the Antagonistic Effect of GA and ABA

Gibberellin and ABA are known to antagonistically regulate various developmental processes throughout the plant life cycle, including stress responses (Yuan et al., 2017), seed germination and seedling growth (Lin et al., 2015; Zhong et al., 2015; Liu et al., 2016), and the formation of arbuscular mycorrhizal symbiosis (Martín-Rodríguez et al., 2016). For example, in rose (*R. hybrida*), the activity of *Rh-PIP2;1*, which is involved in ethylene-mediated rose petal expansion, was reported to be enhanced by GA_3_ at the later stages but suppressed by ABA during the early stages (Ma et al., 2008; Li et al., 2009; Pei et al., 2013). Our previous studies also demonstrated GA and ABA have antagonistic effects on the petal growth in *G. hybrida* (Li et al., 2015), but the nature of the cross-talk between hormones and elongation promoting genes in this system is still not well defined. Findings from the current study suggest that GA and ABA have antagonistic effects on the expression of *GhMIF* which acts as a transcription factor to inhibit petal elongation. GA treatment promotes and ABA suppresses the expression of *GhMIF* with a similar degree. When treated by the biosynthesis inhibitor of endogenous GA and ABA (PAC and FLU), the expression level of *GhMIF* are reversed (**Figure 4B**). These results suggest that the disturbance of endogenous GA and ABA biosynthesis contributes, at least partially, to the antagonistic effect of these two hormones on the expression of *GhMIF* in *G*. *hybrida*.

However, the response of *GEG* to GA and ABA were not congruent with those of GhMIF. *GEG* was activated dramatically by GA application but was suppressed weakly by ABA treatment, which is similar to the expression trend of *GhMIF* with a different extent. One explanation for these observations is that there might be other unknown components that could regulate the expression of *GEG* during GA and ABA signaling pathways. Actually, we also found three other proteins that putatively interact with the *GEG* promoter in the Y1H screen: GhBZR1 (MF370884), GhEIL1 (MF370883), and GhMBF1 (MF370886) (Supplementary Table S2). Based on their sequence homology and annotations in *A. thaliana*, these three proteins may be the components of phytohormone signaling pathways, but whether these proteins influence the expression of *GEG* need to be verified by further researches.

## Conclusion

We present a primary regulation module of GhMIF/*GEG* for ray petal growth of *G. hybrida* in this study. GhMIF, a mini zinc-finger protein, regulates the petal growth by activating the expression of *GEG* through a direct binding to the core region of *GEG* promoter. Although the current data are insufficient to provide a comprehensive view of the role of GhMIF during petal growth due to the unavailability of genomic data of *G. hybrida*, the results generated through this study provide insights into the mechanisms of petal growth regulation and the antagonistic modes of GA and ABA on petal development.

## Author Contributions

MH carried out the experiments and drafted the manuscript. MH and WY supplemented the experiment data for revised manuscript. MH, XJ, and LK analyzed the data. GH, WY, and YT participated in figure preparation. LL and XW participated in experiment design and manuscript revision. YW conceived the study, participated in its design and revised the manuscript. All authors read and approved the final manuscript.

## Conflict of Interest Statement

The authors declare that the research was conducted in the absence of any commercial or financial relationships that could be construed as a potential conflict of interest.

## Footnotes

1 http://www.dna.affrc.go.jp/PLACE/

2 http://bioinformatics.psb.ugent.be/webtools/plantcare/html/

3 http://rsb.info.nih.gov/ij/

4 http://www.dna.affrc.go.jp/PLACE/

5 http://bioinformatics.psb.ugent.be/webtools/plantcare/html/
