# Supplementary material for: A Mini Zinc-Finger Protein (MIF) from Gerbera hybrida Activates the GASA Protein Family Gene, GEG, to Inhibit Ray Petal Elongation
Source: Front Plant Sci. 2017 Sep 22;8:1649. doi: 10.3389/fpls.2017.01649 (PMC5615213; doi:10.3389/fpls.2017.01649)
Supplement: Supplementary file 1 [file Table_1.DOCX]

| **Supplemental Table 1 Primers used in this study.** | | | | | | | | |  |  |
| --- | --- | --- | --- | --- | --- | --- | --- | --- | --- | --- |
|  | | | **Forward primer (5’ to 3’)** | | | **Reverse primer (5’ to 3’)** | | |  |  |
| **The cloning of** GEG promoter | | | | | | | | |  |  |
| LAD1 | | ACGATGGACTCCAGAGCGGCCGC(G/C/A) N(G/C/A) NNNGGAA | | | |  | | |  |  |
| LAD2 | | ACGATGGACTCCAGAGCGGCCGC (G/C/T) N (G/C/T) NNNGGTT | | | |  | | |  |  |
| LAD3 | | ACGATGGACTCCAGAGCGGCCGC(G/C/A) (G/C/A) N(G/C/A) NNNCCAA | | | |  | | |  |  |
| LAD4 | | ACGATGGACTCCAGAGCGGCCGC(G/C/T) (G/A/T) N(G/C/T) NNNCGGT | | | |  | | |  |  |
| AC1 | | ACGATGGACTCCAGAG | | | |  | | |  |  |
| RB-0a | |  | | | | GACAGCGGAATGGTTGATAAAATG | | |  |  |
| RB-1a | |  | | | | ACGATGGACTCCAGTCCGGCCCCATGATATTGTAACCTTTGCCTCA | | |  |  |
| RB-2a | |  | | | | TTCATTAAACACCGAGCGATTAAAGT | | |  |  |
| **GUS assay** | | | | | | | | |  |  |
| *P1365-GUS* | | | GAATTCACACCCCAAAAGCAACCATCTA | | | GGATCCTTTGTTTTGGGTTGAGTTTGTGC | | |  |  |
| *P880-GUS* | | | GAATTCAGCACTGATCTCGTGATACCTATG | | | GGATCCTCTTTGTTTTGGGTTGAGTTTGT | | |  |  |
| *P580-GUS* | | | GAATTCCCTAAAATTTGTGTTCATATGCAGC | | | GGATCCTTGTTTTGGGTTGAGTTTGTGC | | |  |  |
| *P260-GUS* | | | GAATTCATTATTTGCAGTTAAGAGGTCATGAG | | | GGATCCTCTTTGTTTTGGGTTGAGTTTGT | | |  |  |
| **Dual-luciferase assay** | | | | | | | | |  |  |
| *pGreenII-0800-P1365* | | | | CTCGAGACACCCCAAAAGCAACCATCTA | | | GGATCCTTTGTTTTGGGTTGAGTTTGTGC | | |  |
| *pGreenII-0800-P880* | | | | CTCGAGAGCACTGATCTCGTGATACCTATG | | | GGATCCTCTTTGTTTTGGGTTGAGTTTGT | | |  |
| *pGreenII-0800-P580* | | | | CTCGAGCCTAAAATTTGTGTTCATATGC | | | GGATCCTTGTTTTGGGTTGAGTTTGTGC | | |  |
| *pGreenII-0800-P260* | | | | CTCGAGATTATTTGCAGTTAAGAGGTCAT | | | GGATCCTCTTTGTTTTGGGTTGAGTTTGT | | |  |
| *pGreenII-0800*-  pGEG_320_ | | | | CTCGAGAACCTAAAATTTGTGTTCATATGCA | GGATCCGGTTGTTATCCTTGTTATCCTTTTT | | | | | |
| *PGreenII-0800-*  *pGEG_170_*  *pBS-GhMIF* | CTCGAGTACATGAGCCACTACGTTTGATA  GGATCCAGAAAGACACCTCTCTAAAGAATGACA | | | | GGATCCGATGATTAAACGGTTGTTATCCT  TCTAGACACAAAACCCCATTGCTTCTAA | | |  |  |  |
| **Yeast one-hybrid assays** | | | | | | | | |  |  |
| GhMIF-AD  pAbAi-pGEG_170_ | | | GAATTCAGAAAGACACCTCTCTAAAGAATGACA  GAGCTCTACATGAGCCACTACGTTTGATA | | | CTCGAGCACAAAACCCCATTGCTTCTAA  CTCGAGGATGATTAAACGGTTGTTATCCT | | |  |  |
| pAbAi-pGEG_320_ | | | GAGCTCAACCTAAAATTTGTGTTCATATGCA | | | CTCGAGGGTTGTTATCCTTGTTATCCTTTTT | | |  |  |
| **EMSA** | | | | | | | | |  |  |
| GhMIF  *pGEG_150_*  *pGEG_170_* | | | GGATCCAGAAAGACACCTCTCTAAAGAATGACA  AACCTAAAATTTGTGTTCATATGC  TACATGAGCCACTACGTTTGATA | | | GAATTCCACAAAACCCCATTGCTTCTAA  TAAAACGTGTTCTTGGGTGACTT  GATGATTAAACGGTTGTTATCCT | | |  |  |
| *pGEG_320_* | | | AACCTAAAATTTGTGTTCATATGC | | | TAAAACGTGTTCTTGGGTGAC | | |  |  |
| **Subcellular location assay** | | | | | | | | |  |  |
| YFP-GhMIF | | | GAATTCAGAAAGACACCTCTCTAAAGAATGACA | | | AAGCTTCACAAAACCCCATTGCTTCTAA | | |  |  |
| **Transactivation assay** | | | | | | | | |  |  |
| GhMIF-BD | | | GAATTCAGAAAGACACCTCTCTAAAGAATGACA | | | GGATCCCACAAAACCCCATTGCTTCTAA | | |  |  |
| **Transient transformation assays** | | | | | | | | |  |  |
| *pTRV2-GhMIF* | | | GAATTCAGAAAGACACCTCTCTAAAGAATGACA | | | GGATCCCACAAAACCCCATTGCTTCTAA | | |  |  |
| *pCANG-GhMIF* | | | GGATCCGCATGACAAAGAGACGAGTGGTGTTGA | | | GAGCTCCTAAGGTGAAGACGGTTCAGTTTC | | |  |  |
| **qRT-PCR** | | | | | | | | |  |  |
| *GhMIF* | | | AGACGAGTGGTGTTGAAGAGGGA | | | ACTCACCGTATCTCACATGAACCC | | |  |  |
| *GEG* | | | GCCTTTTCTTGCTTTTGCTCTTC | | | CGCCTCATCAATCTTGTTCACC | | |  |  |
| *GhEXP* | | | CCTTGTTGACTGTTGCTGTTGTTG | | | TTCTACGGATTAACTTTCACCACCC | | |  |  |
| *GhEXP1* | | | GGGTTACGGTACAAACACTGCG | | | TGGGGTCGTCATTGCATCTCAT | | |  |  |
| *GhEXP3* | | | TTAAGCTGTGGGTCATGCTACGA | | | GCGTTGTTAGGTAAGGCGTTGTT | | |  |  |
| *GhEXP10* | | | TGGCAAAGCAACTCTTACCTCAAC | | | CCCTTGGAATGTCTGACCAAACTG | | |  |  |
| *GhTUB2* | | | TCAAGATGCAGTCACTGATGATGTC | | | CATACACGATACCTGAACTACACGC | | |  |  |
| *GhCESA* | | | GGAATGTACCTCTGGCCAACATGA | | | CTCCCACAACGCGTTTTGGCCCAT | | |  |  |
| *GhXET* | | | GTGTACGCACATGGAAAGGGTG | | | ATGGTGGTTCCAGAGGATGGTG | | |  |  |
| *GhACTIN* | | | CGAGGCACTGGTATTGTGTTGG | | | AGGGAATCTGTAAGGTCACGCC | | |  |  |
