# Supplementary material for: A Mini Zinc-Finger Protein (MIF) from Gerbera hybrida Activates the GASA Protein Family Gene, GEG, to Inhibit Ray Petal Elongation
Source: Front Plant Sci. 2017 Sep 22;8:1649. doi: 10.3389/fpls.2017.01649 (PMC5615213; doi:10.3389/fpls.2017.01649)
Supplement: Supplementary file 2 [file Table_2.DOCX]

#### Supplementary Table 2 Screening results of interaction proteins with *GEG* promoter.

| Accession number | Annotation (*A. thaliana*) | | Coverage | | Identity | | Clone No | |  |
| --- | --- | --- | --- | --- | --- | --- | --- | --- | --- |
| Hormone-associated (4) | | | | | | | | |  |
| MF370883 | | protein ethylene insensitive 3 /ethylene insensitive 3-like 1 | | 88% | | 77% | | 2 | |
| MF370886 | | multiprotein-bridging factor 1b (MBF1) /ethylene-responsive transcriptional coactivator | | 92% | | 81% | | 2 | |
| MF370884 | | BES1/BZR1 homolog 4/BES1/BZR1-like protein 3 | | 76% | | 69% | | 1 | |
| MF370885 | | mini zinc finger 2 | | 95% | | 69% | | 1 | |
