# Supplementary material for: A Mini Zinc-Finger Protein (MIF) from Gerbera hybrida Activates the GASA Protein Family Gene, GEG, to Inhibit Ray Petal Elongation
Source: Front Plant Sci. 2017 Sep 22;8:1649. doi: 10.3389/fpls.2017.01649 (PMC5615213; doi:10.3389/fpls.2017.01649)
Supplement: Supplementary file 3 [file Presentation_1.PDF]

Supplemental Materials and Methods

Plant Materials and Growth Conditions

Mature seeds of (*Oryza sativa* L., cv. Zhonghua 11) were dehusked and surface sterilized with 70% (v/v) ethanol for 2 min and sodium hypochlorite (NaOCl) for 5 min, then washed 5 times with sterile distilled water. The seeds were placed on callus induction medium (MS containing 0.5 mgL^{-1} 2,4-dichlorophenoxy acetic acid, 1.0 mgL^{-1} kinetin, pH 5.8) in Petri dishes, which were then sealed with Parafilm (PARAFILM, USA) and incubated at 25 ± 2 ℃ in the dark for 10 d, after which the seeds were transferred to fresh medium. After another 21 d, friable calli were sub-cultured on fresh medium for transformation.

Transformation of Rice Callus and GUS Staining

The P1365, P880, P580 and P260 sequences fused with GUS in the pBI101 vector were transformed into *Agrobacterium tumefaciens* strain EHA105 by electroporation. Suitable sized calli (3-5 mm) were immersed in an *A. tumefaciens* suspension (OD600=0.6) containing 50 μM acetosyringone (4-acetyl-2, 6-dimethoxyphenol). Infected calli were blot-dried and kept on MS medium for cultivation at 26 ℃ in the dark for 3 d, then washed with sterile distilled water containing 500 mg/L cefotaxime for 10 min. Non-transformed calli were used as a control. A GUS assay was performed using a X-glucuronide solution (0.01 M $NaPO_4$, pH 7.0, 1 mM EDTA, 10 mM X-glucuronide, and 1% [v/v] Triton-X100). Transgenic and control calli were completely immersed in the X-glucuronide solution and incubated at 37 ℃ for 12-15 h in the dark, followed by overnight soaking in 70% ethanol to remove pigments. The samples were observed and photographed using an Olympus BX Microscope (Olympus Corporation, Japan).
