# Supplementary material for: A Mini Zinc-Finger Protein (MIF) from Gerbera hybrida Activates the GASA Protein Family Gene, GEG, to Inhibit Ray Petal Elongation
Source: Front Plant Sci. 2017 Sep 22;8:1649. doi: 10.3389/fpls.2017.01649 (PMC5615213; doi:10.3389/fpls.2017.01649)
Supplement: Supplementary file 4 [file Data_Sheet_1.DOCX]

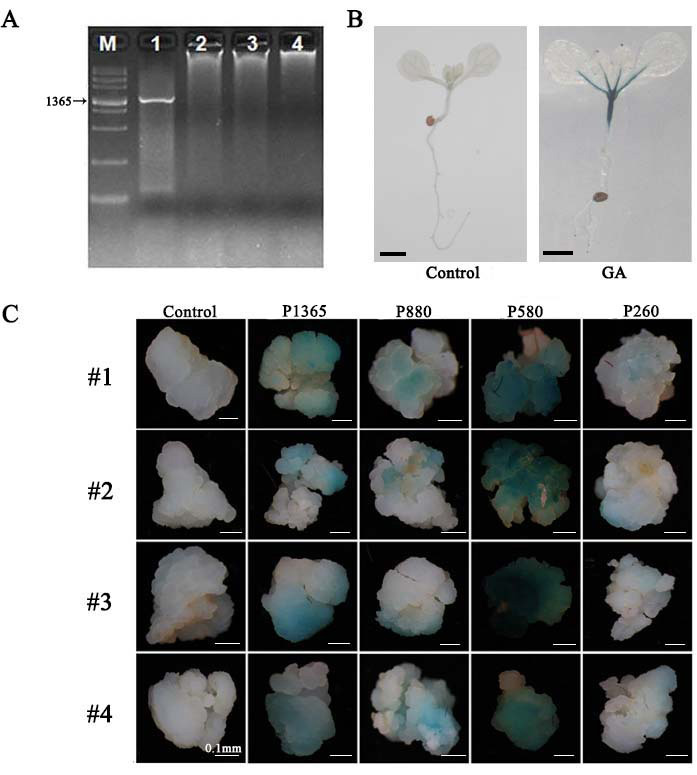


**Supplemental Figure 1 Cloning and activity analysis of *GEG* promoter.** (A) PCR products of the secondary amplification of Hi-TAIL PCR. The 1365bp of *GEG* promoter was amplified using the gene specific primer, RB-2a, and the 16-mer primer, AC1. (B) Histochemical staining of *p1365::GUS* transgenic seedlings in response to gibberellic acid (GA) (n=10, 10 seedlings from each genotype were used). (C) Histochemical staining of transformed rice calli carrying different promoter sequences (P1365, P880, P580, P260) (n=4, 4 calli from each genotype were used). Scale bar represents 200μm (B) or 0.1mm (C).

**
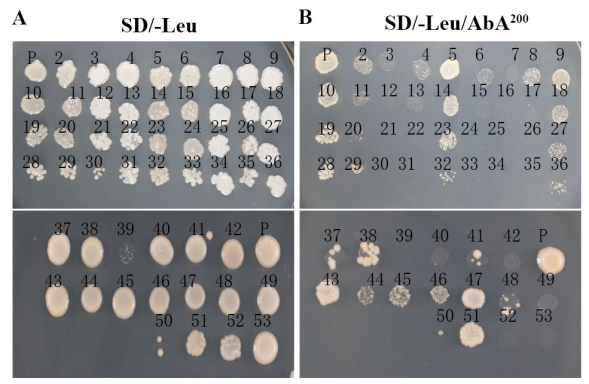
**

**Supplemental Figure 2** **Screening results of proteins interacting with the *GEG* promoter.** (A) A total of 53 positive clones grown on the SD/-ura/-Leu/ medium. (B) Seventeen positive clones grown on the SD/-ura/-Leu/AbA^200^ medium. Vectors pAbAi-53 + pGADT7- Rec T were introduced into yeast as positive controls (P).


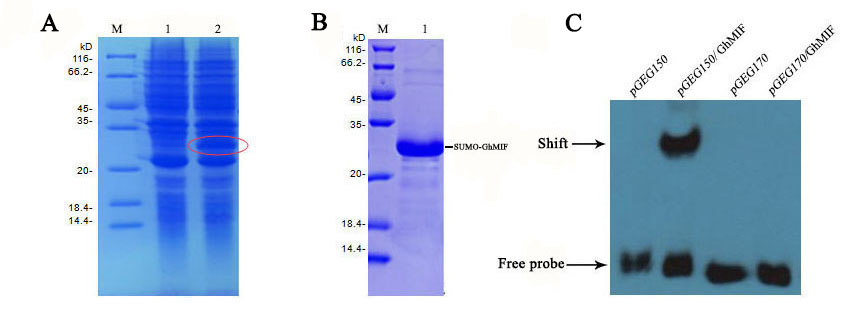


**Supplemental Figure 3**: **The protein purification and interaction of GhMIF and *GEG* promoter.** (A) Prokaryotic expression and (B) purification of recombinant SUMO-GhMIF protein in *Escherichia coli* on SDS-PAGE. (A) Lane 1-2: total protein from *E. coli* cells containing pET28a-SUMO-GhMIF before (Lane 1) and after (Lane 2) induction with IPTG. The red circle represents the location of the fused protein, SUMO-GhMIF. (B) Lane 1: the purified SUMO-GhMIF protein. The migration of molecular mass (M) standards is given at the left in KD. (C) EMSA assay confirming the interaction of GhMIF and *pGEG_150_* not *pGEG_170_*.


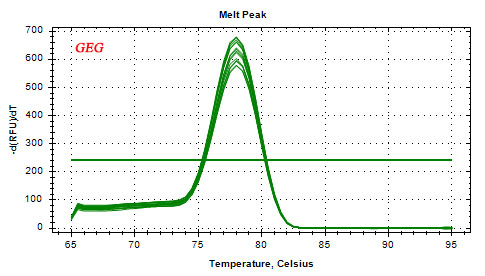


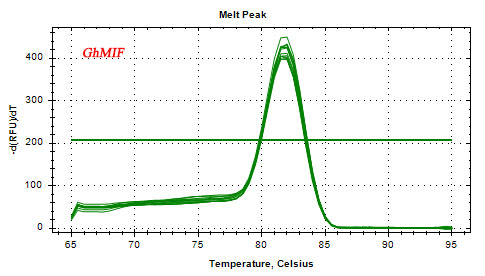


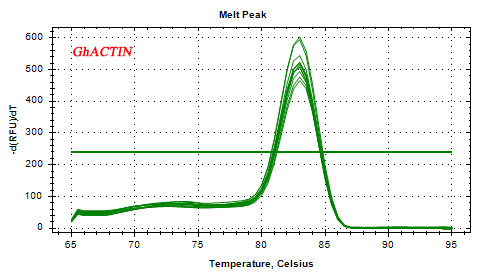


**Supplemental Figure 4 Melting curves for the target and reference genes.**
